# Wicked Problems: An Exploration of Elder Mistreatment Complexity

**DOI:** 10.1093/geroni/igaa057.109

**Published:** 2020-12-16

**Authors:** Julia Rowan, Kelly Marnfeldt, Kathleen Wilber, Susan Enguidanos

**Affiliations:** 1 University of Southern California, Oxnard, California, United States; 2 University of Southern California, Santa Monica, California, United States; 3 University of Southern California, Chatsworth, California, United States; 4 University of Southern California, Los Angeles, California, United States

## Abstract

Elder mistreatment (EM) complexity, while described anecdotally, lacks an empirical foundation for measurement. Improved knowledge on the range and nature of concurrent issues that complicate EM intervention would inform the development of more effective solutions and enable greater precision of evaluation research. The purpose of this qualitative study was to explore factors contributing to complexity in a sample of EM cases that was selected based on difficulty reaching resolution. The sample was drawn from those reviewed by an experienced EM Multidisciplinary team (MDT) and determined to require long-term case management (n=39) beyond the capacity of the MDT’s usual response. Case manager narrative documentation of ongoing assessment and social service records were qualitatively coded by two researchers. Inductive content analysis, with iterative code reconciliation, was used to identify issues and problems both related and concurrent to EM. Eighteen themes and 74 sub-themes emerged, with 93% initial coding agreement between researchers. The most frequent themes were problems with Caregiving (80%), Cognition (80%), Physical Health (80%), Behavioral Health (69%), Socialization (64%), and Finances (62%). Refusal of formal services was common (90%), yet all accepted visitation by the case manager, suggesting informal support may be effective. Diversity, interconnectedness, and emergence of issues along the duration of case management indicates a system approach to intervention design and evaluation is warranted. This research underscores the need for holistic intervention for highly complex EM, and lays the foundation for objective measure of complexity to standardize selection for specialized intervention.

